# Corrigendum: Hip Development Dysplasia – Part 1

**DOI:** 10.1055/s-0044-1785685

**Published:** 2024-04-10

**Authors:** Susana Reis Braga, Amâncio Ramalho Júnior, Miguel Akkari, Marina Juliana Pita Sassioto Silveira Figueiredo, Gilberto Waisberg, Claudio Santili

**Affiliations:** 1 Médico assistente, Grupo de Ortopedia Pediátrica, Departamento de Ortopedia e Traumatologia, Santa Casa de Misericórdia de São Paulo, São Paulo, SP, Brasil; 2 Médico assistente, Grupo de Ortopedia Pediátrica, Hospital Israelita Albert Einstein, São Paulo, SP, Brasil; 3 Médica ortopedista e traumatologista, Hospital Maria Aparecida Pedrossian, Universidade Federal de Mato Grosso do Sul, Campo Grande, MS, Brasil; 4 Médico ortopedista e traumatologista, Grupo de Ortopedia e Traumatologia Pediatrica, Hospital Mario Covas, Faculdade de Medicina do ABC, Santo André, SP, Brasil

**Corrigendum**

Dear readers,

In the article “Developmental Dysplasia of the Hip – Part 1” (Rev Bras Ortop 2023;58(6):e839–e846. Doi 10.1055/s-0042-1758371 .), published online in *Rev Bras Ortop* in December 2023,

**Where it reads:**

developmental dsysplasia of the hip

**It should read:**

developmental dysplasia of the hip

