# Supplementary material for: Corrigendum: Hip Development Dysplasia – Part 1
Source: Rev Bras Ortop (Sao Paulo). 2024 Apr 10;59(2):e336. doi: 10.1055/s-0044-1785685 (PMC12135050; doi:10.1055/s-0044-1785685)
Supplement: Related texts

# Correction: Corrigendum: Displasia do desenvolvimento do quadril – Parte 1

**Corrigendum**

Caros leitores,

No artigo “Displasia do desenvolvimento do quadril – Parte 1” (Rev Bras Ortop 2023;58(6):e839–e846. Doi 10.1055/s-0042-1758371 .), publicado on-line na *Rev Bras Ortop* em dezembro de 2023,

**Onde se lê:**

developmental dsysplasia of the hip

**Deveria ser:**

developmental dysplasia of the hip
